# Prenatal exposure to polybrominated diphenyl ethers and inattention/hyperactivity symptoms in mid to late adolescents

**DOI:** 10.3389/fepid.2023.1061234

**Published:** 2023-06-21

**Authors:** Seonyoung Park, Whitney Cowell, Amy E. Margolis, Andreas Sjodin, Richard Jones, Virginia Rauh, Shuang Wang, Julie B. Herbstman

**Affiliations:** ^1^ Department of Environmental Health Sciences, University of Michigan School of Public Health, Ann Arbor, MI, United States; ^2^ Department of Environmental Health Sciences, Columbia University Mailman School of Public Health, New York, NY, United States; ^3^ Departments of Pediatrics and Population Health, NYU Grossman School of Medicine, New York, NY, United States; ^4^ Department of Psychiatry, Columbia University, New York, NY, United States; ^5^ Division of Laboratory Sciences, National Center for Environmental Health, Centers for Disease Control and Prevention, Atlanta, GA, United States; ^6^ Department of Population and Family Health, Columbia University Mailman School of Public Health, New York, NY, United States; ^7^ Department of Biostatistics, Columbia University Mailman School of Public Health, New York, NY, United States

**Keywords:** Polybrominated diphenyl ethers (PBDE), inattention, hyperactivity, adolescents, prenatal exposure, sex-specificity

## Abstract

**Introduction:**

Prenatal exposure to polybrominated diphenyl ethers (PBDEs) has been associated with increased symptoms of attention deficit/hyperactivity disorder (ADHD) in early to middle childhood, as well as early adolescence. However, data are limited for the long-lasting impact of exposure on outcomes assessed across the entire adolescent period and the sex-specificity of such associations.

**Methods:**

We investigated the association between continuous natural-log-transformed cord plasma PBDE concentrations and ADHD rating scale 4th edition (ADHD-RS-IV) score from mid adolescence (approximately 11 years old) to late adolescence (approximately 17 years old). The study sample includes a subset (*n* = 219) of the African American and Dominican children enrolled in the Columbia Center for Children's Environmental Health Mothers and Newborns birth cohort. We used generalized estimating equations to account for the repeated measure of ADHD-RS scores. We examined interactions between exposure to PBDE and sex using cross-product terms and sex-stratified models. In addition, we used linear regression using an age-stratified sample as a sensitivity analysis.

**Results and Discussion:**

Associations between prenatal exposure and parents’ reports of ADHD symptoms varied by sex (*p*-interaction <0.20), with positive relationships observed among girls but not boys from sex-stratified models. Our finding suggests prenatal exposure to PBDE may affect ADHD symptoms assessed during middle to late adolescence and the sex-specificity of such impact. Our results can be confirmed by future studies with larger and more diverse samples.

## 1. Introduction

Polybrominated diphenyl ethers (PBDEs) are a class of chemicals that were widely used as flame retardants in various household products, such as furniture foam padding, rugs, electronics, and industrial products, including plastics, building materials, and textiles. While penta- and octa-BDE (mixtures containing five and eight bromine atoms, respectively) were phased out in the United States beginning in 2004, existing household products and the recycling of PBDE-containing products remain sources of ongoing human exposure (1, 2). Since PBDEs are not chemically bound to base polymers, they can easily migrate from products and bioaccumulate up the food chain, providing a persistent source of exposure (2, 3). Children's exposure to PBDE can occur through multiple exposure pathways, including transfer across the placenta, breastfeeding, or household dust. Although PBDE levels have decreased in the US population since their phase-out, their ubiquity and high environmental persistence has led to the continued detection of concentrations in the blood of mothers and children (4).

Mounting evidence from epidemiological studies has demonstrated that PBDEs are associated with diabetes, cancer, reproductive health effects, and altered thyroid function (5, 6). PBDE exposure during fetal development is negatively associated with children's neurodevelopment (7). In particular, prenatal exposure to PBDE has been associated with poor executive function, including attention deficit/hyperactivity disorder (ADHD) and attention-related behavioral problems (8). These disorders have been shown to negatively affect academic and vocational achievement and are often accompanied by other comorbidities, such as substance abuse, alcohol abuse, or depression (9–11). Previous studies suggest that prenatal exposure to PBDEs may be associated with the development of symptoms of ADHD at various ages from early childhood through early adolescence (12–14). However, few studies have assessed whether these PBDE-associated symptoms of ADHD persist into mid/late adolescence. The aim of the present study was to extend prior research by examining the long-term effect of prenatal PBDE exposure on children's attention-related behaviors in mid/late adolescence and examining the sex difference in such associations. Sex differences are critically important to consider as they help understand the susceptibility and etiology of ADHD, and the mechanisms by which PBDE exposure raises the risk of ADHD.

## 2. Materials and methods

### 2.1. Study design and participants

**Figure 1:**
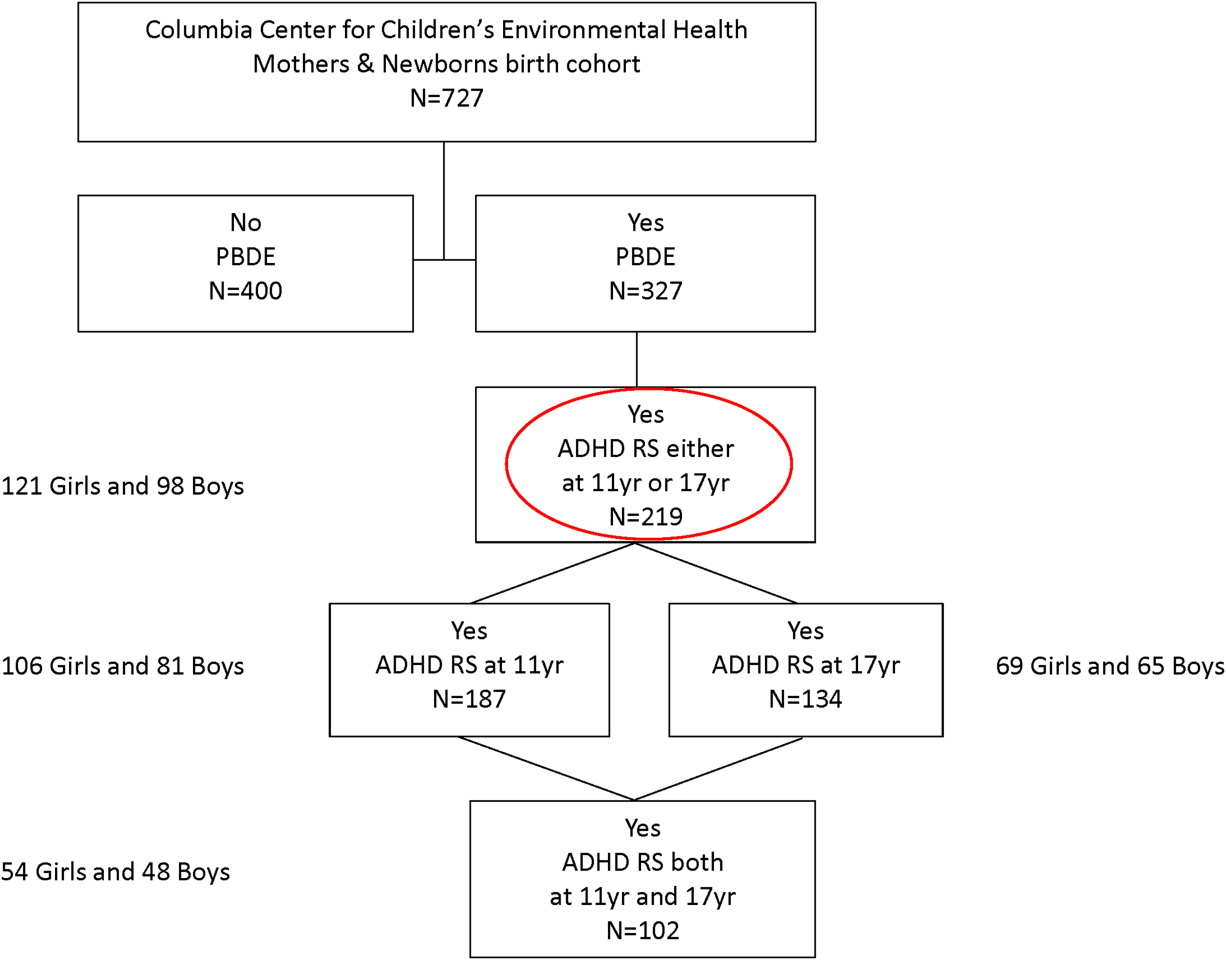
Diagram of study enrollment and follow-up.

This analysis includes 219 of the 727 children enrolled in the Columbia Center for Children's Environmental Health (CCCEH) Mothers and Newborns birth cohort (Figure 1). The cohort was originally designed to examine various health effects associated with exposure to prenatal and postnatal environmental chemicals. Women living in northern Manhattan or the south Bronx between 1998 and 2006 were enrolled in the study and followed prospectively (15). The inclusion criteria include non-active smokers during pregnancy and free of diabetes, hypertension, HIV, and drug abuse (15). Of the 727 children enrolled, 219 had both cord plasma PBDE concentrations measured at birth and ADHD Disorder Rating Scale (ADHD-RS-IV) measured at either approximately 11 years (range 9–14 years) or 17 years (range 14–21 years) of age. Table 1 presents the demographic and lifestyle characteristics of the study sample and those who were excluded from our analysis. In addition, demographics in two age groups (∼11 vs. ∼17 years) are summarized in Supplementary Table S1.

**Table 1:** Characteristics of participants with plasma PBDE concentrations and ADHD rating scale (*n* = 219) and comparison with excluded group (*n* = 508).

	Excluded sample (*n* = 508)	Included sample (*n* = 219)	Girls (*n* = 121)	Boys (*n* = 98)
Maternal characteristics				
Age (years)	24.62 ± 4.89	24.97 ± 5.01	25.08 ± 5.13	24.84 ± 4.88
Education				
<High school	27 (2.17)	10 (4.57)	5 (4.13)	5 (5.10)
High school or GED	352 (69.29)	153 (69.86)	85 (70.25)	68 (69.39)
≥College degree	118 (23.23)	53 (24.20)	28 (23.14)	25 (25.51)
Married/living with partner	136 (26.77)	58 (26.48)	30 (24.79)	28 (28.57)
Race/ethnicity				
African American	163 (32.09)	91 (41.55)	53 (43.80)	38 (38.78)
Dominican	345 (67.91)	128 (58.45)	68 (56.20)	60 (61.22)
Non-verbal intelligence	86.17 ± 13.17	84.35 ± 13.37	84.70 ± 13.36	83.93 ± 13.44
Household characteristics				
Material hardship^a^	203 (39.96)	87 (39.72)	52 (42.97)	35 (35.71)
Smoker in home	173 (34.05)	73 (33.33)	44 (36.36)	29 (29.59)
Child characteristics				
Birth weight (kg)	3.33 ± 0.48	3.44 ± 0.53	3.38 ± 0.59	3.50 ± 0.44
Gestational age (weeks)	38.43 ± 6.53	39.12 ± 3.52	39.34 ± 1.27	38.85 ± 5.07
Breastfed ≥12 weeks^b^	162 (31.89)	75 (34.25)	37 (30.58)	38 (38.77)
Cord PBDE (ng/g lipid)^c^				
BDE 47	15.07 ± 3.02	13.26 ± 3.07	13.17 ± 3.02	13.38 ± 3.15
BDE 99	4.01 ± 2.66	3.62 ± 2.47	3.60 ± 2.47	3.65 ± 2.49
BDE 100	3.45 ± 2.27	2.90 ± 2.10	2.81 ± 2.06	3.02 ± 2.15
BDE 153	2.91 ± 1.75	2.57 ± 1.73	2.42 ± 1.65	2.75 ± 1.80

PBDE, polybrominated diphenyl ether; ADHD, attention deficit/hyperactivity disorder.

Values are mean ± SD or *n* (%). All characteristics are measured at baseline (pregnancy/delivery) unless otherwise noted.

^a^ Self-reported inability to afford adequate access to food, clothing, or housing.

^b^ Percent of “Yes” for breastfeed ≥12 weeks.

^c^ Geometric mean.

All study protocols were approved by the Institutional Review Board (IRB) of Columbia University and the Centers for Disease Control and Prevention (CDC) deferred its review to the Columbia University IRB. Mothers were informed about all study procedures before each visit and provided written informed consent to participate. Children provided informed assent beginning at the age of 7 years.

### 2.1. ADHD rating scale-IV

The ADHD-RS-IV home version was used to assess the severity of inattention and hyperactivity/impulsivity symptoms based on parents’ reporting (16). The ADHD-RS was developed based on the diagnostic criteria for ADHD as described in the Diagnostic and Statistical Manual of Mental Disorders 4th edition (16, 17). Parents rated their children's behavior over the past 6 months, which was used as a “current rating” of symptoms, and over the child's entire lifetime, excluding the past 6 months, which was used as a “worst ever” rating of symptoms. The questionnaire consists of 18 questions: half query inattentive symptoms and half query hyperactive symptoms, and the answer for each question depends on the frequency of symptoms (0 = never or rarely, 1 = sometimes, 2 = often, and 3 = very often). Thus, the ADHD-RS-IV yields two subscale scores: (1) the Inattention subscale score; and (2) the Hyperactivity-Impulsivity subscale score, and each subscale score is in the range of 0–27. Then, the Total Scale score (range 0–54) is calculated by summing the Inattention and Hyperactivity-Impulsivity subscale scores. A spaghetti plot showing the change of ADHD-RS scores of children whose scores were reported twice are presented in Supplementary Figure S1. For the main analyses, we used the three scores (Inattention, Hyperactivity-Impulsivity, Total) based on the parent's “current rating” of symptoms. In the secondary analyses, we further explored associations with the “worst ever” scores. In our analysis, continuous measures of ADHD-RS-IV scores were used as guided by the Research Domain Criteria of the National Institute of Mental Health, where mental health and psychopathology are considered in the context of major domains of neurobehavioral functioning, unlike traditional category-based diagnostics (18).

### 2.3. PBDE exposure assessment

At delivery, umbilical cord plasma was collected by research staff, and transported and stored at −70°C at the CCCEH laboratory. An analysis of stored samples was conducted at the CDC for the measurement of 11 PBDE congeners (BDEs: 17, 28, 47, 66, 85, 99, 100, 153, 154, 183, and 209). In the present analysis, we examined the four most frequently detected PBDE congeners: BDEs-47 (70.4%), 99 (48.4%), 100 (37.8%), and 153 (33.7%). Briefly, cord plasma samples were fortified with internal standards, and plasma samples were extracted using a Gilson 215 liquid handler (Gilson Inc., Middleton, WI, United States). Plasma PBDE concentrations were measured using gas chromatography isotope dilution high-resolution mass spectrometry on a DFS instrument (ThermoFisher, Bremen, Germany) (19, 20). Blanks (*n* = 3) were measured for every 30 samples; then the median blank value was subtracted from the measured sample concentration to obtain the final PBDE concentration. In addition, lipids were removed using a rapid trace modular SPE workstation (Biotage, Uppsala, Sweden), and total cholesterol and triglycerides were measured using commercial test kits (Roche Diagnostics, Indianapolis, IN, United States).

Total plasma lipids were estimated from these measured components using the short formula described by Phillips et al. (21), and the total cord blood lipid levels, including unmeasured free cholesterol and phospholipids, were estimated by the summation of individual lipid components using an umbilical cord blood–specific formula (total cord blood lipids = 2.657 × total cord blood cholesterol + cord blood triglycerides +0.268, in g lipids/L plasma; Sjodin A, personal communication). PBDEs were examined as a lipid-standardized variable in all models (ng/g lipid). Detailed information regarding the measurement of PBDE concentrations in this cohort has been previously published (22).

### 2.4. Statistical analysis

We substituted values below the limit of detection (LOD) by LOD divided by the square root of 2 (22). Then, we used multivariable linear regression to examine the association between log-transformed cord plasma PBDE concentrations as linear predictors (separately for BDE-47, 99, 100, and 153) and with the continuous measure of ADHD-RS-IV scores as the outcome of interest. Covariates were selected based on *a priori* knowledge and impact on the main effect estimate (≥10%). We considered demographic/cultural factors, indicators of socioeconomic status, physical characteristics, and maternal intelligence status. Specifically, the models were adjusted for ethnicity (African American/Dominican), parity (multiparous/nulliparous), cord plasma cotinine level—an indicator of cigarette smoke exposure (23), maternal education at delivery (less than high school, high school degree or equivalent, and college level education or higher), material hardship during pregnancy—defined as limited access to basic needs (food, housing, and clothing)—reported through a questionnaire, breastfeeding history (continuous, in weeks), maternal non-verbal intelligence (continuous TONI-II quotient), child’s sex, and child’s exact age at outcome assessment. A similar approach was used in previous studies (24–28). The positive effects of breastfeeding on child behavior and cognitive development have been reported previously (29) and recent studies suggest the potential positive influence of breastfeeding on neurotoxic effect PBDE (30). We included breastfeeding history in our model, although it is likely not directly associated with prenatal exposure, since it may explain some of the variance associated with the outcome and/or indirectly influence the effect of prenatal PBDE exposure on ADHD symptoms through backdoor paths.

We used generalized estimating equations (GEE) with an exchangeable correlation structure to account for the repeated measure of ADHD-RS scores measured at approximately 11 years old and/or 17 years old (31).

To explore the potential sex-specific associations between prenatal exposure to PBDE and children's symptoms of ADHD, we included an interaction term of natural-log-transformed cord plasma PBDE concentration and sex assigned at birth. We used the Wald test statistic for the regression coefficient of the interaction term (PBDE * sex) to evaluate its statistical significance. As used in previous studies (32–34), we used a *p*-value <0.20 for the interaction term as a threshold to consider possible effect modification by children's sex, acknowledging that our statistical power for these analyses was limited. When the interaction term was significant at a significance level of 0.20, additional analyses were conducted stratifying by sex.

In the sensitivity analyses, we examined whether the symptoms of ADHD were sensitive to exposure trajectories as described previously (35). To briefly explain, the PBDE exposure trajectories from birth through the age of 9 years were determined by using a latent class growth analysis (LCGA) in previous study (35) and we examine the effect of membership in each trajectory (Persistent low vs. Early postnatal peak vs. Prenatal high vs. Sustained postnatal high) on the continuous measure of ADHD-RS-IV scores. In addition, we repeated the main regression modeling using wet-weight PBDEs with cord blood cholesterol and cord blood triglycerides as covariates in the model. This result was compared with lipid-standardized PBDE modeling results.

## 3. Results

### 3.1. Study participants

At the time of enrollment, all mothers self-identified as Dominican (58%) or African American (42%). At delivery, the mothers had a mean age of 24.9 years, approximately 74% had a high school education or less, 26% were married or living with a partner, and 33% lived with a smoker in the home. One-third of mothers (34.2%) reported that they breastfed the child participating in the study for 12 weeks or longer. The mean ± standard deviation (SD) of maternal TONI-II non-verbal intelligence test quotient was 84.3 ± 13.3. We did not observe any significant difference in demographics and PBDE levels between the age groups (∼11 vs. ∼17 years) based on a one-way ANOVA or chi-square test. We observed that African American women had higher geometric mean cord plasma concentrations of BDE-47 (20.53 ± 3.03 vs. 10.64 ± 2.87, *p* < 0.01), BDE-99 (5.15 ± 2.57 vs. 2.98 ± 2.23, *p* < 0.01), BDE-100 (3.66 ± 2.26 vs. 2.56 ± 1.92, *p* < 0.01), and BDE-153 (2.73 ± 1.86 vs. 2.50 ± 1.64, *p* = 0.06) compared to Dominican women, and a similar trend was reported in the larger population (4). Additional characteristics of the study participants are presented in Table 1 and Supplementary Table S1.

As briefly mentioned above, BDE-47, 99, 100, and 153 were the most frequently detected congeners. The geometric mean concentration was highest for BDE-47 (13.2 ng/g lipid), followed by BDE-99 (3.6 ng/g lipid), BDE-100 (2.9 ng/g lipid), and BDE-153 (2.5 ng/g lipid).

### 3.2. Sex differences in PBDE concentrations and ADHD rating scales

No significant sex differences were observed between cord plasma PBDE concentrations, with the exception that boys have a higher trend level concentration of BDE-153 compared to girls (*p* = 0.06). Overall and sex-specific geometric mean concentrations of cord plasma PBDE concentrations are presented in Table 1.

**Figure 2:**
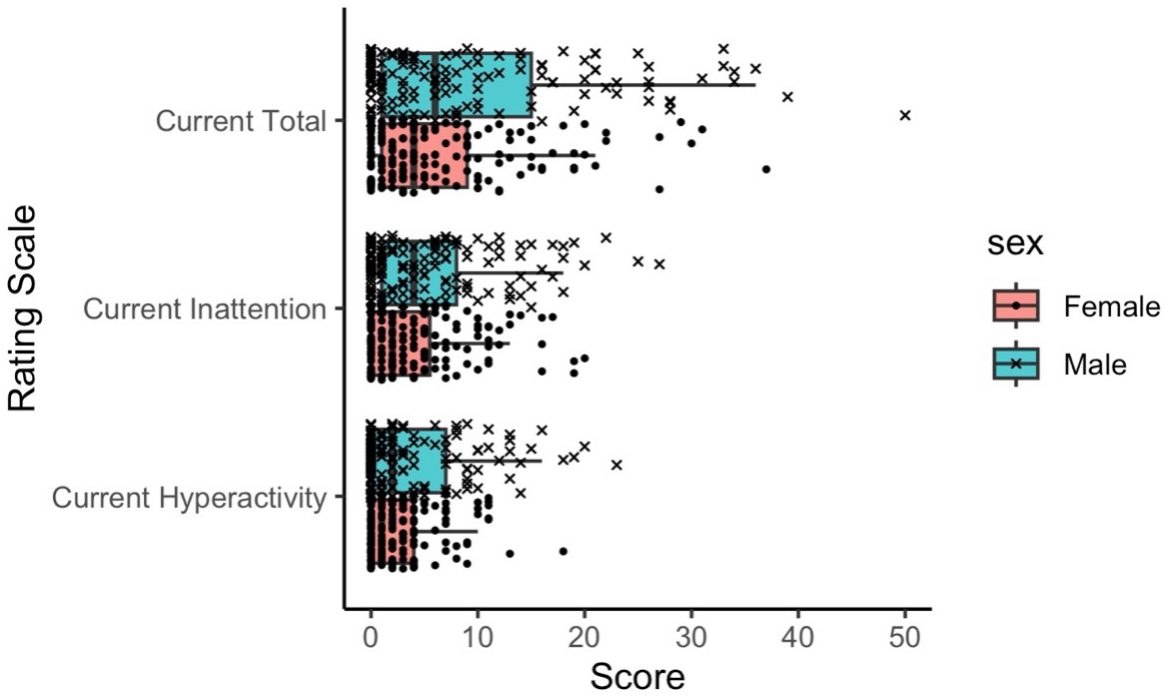
Distribution of ADHD rating scale scores for each sex. Each subscale score (Hyperactivity or Inattention scale) ranges from 0 to 27, while the Total scale score ranges from 0 to 54. ADHD, attention deficit/hyperactivity disorder.

As shown in Figure 2, we found significant differences between boys and girls on all three ADHD-RS scores (*p* ≤ 0.01), with boys having higher mean SD scores: Current Inattention (5.61 ± 5.94 vs. 3.75 ± 4.56); Current Hyperactivity-Impulsivity (3.80 ± 4.97 vs. 2.57 ± 3.24); and Current Total (9.38 ± 10.40 vs. 6.25 ± 7.28).

As hypothesized, the interaction terms of sex and cord plasma PBDE concentrations have a *p*-value < 0.20. Therefore, we regarded sex as a potential effect modifier in the association between prenatal exposure to PBDE and symptoms of ADHD and ran sex-stratified models as the main analysis.

### 3.3. Exposure to PBDE and ADHD rating scales

**Figure 3:**
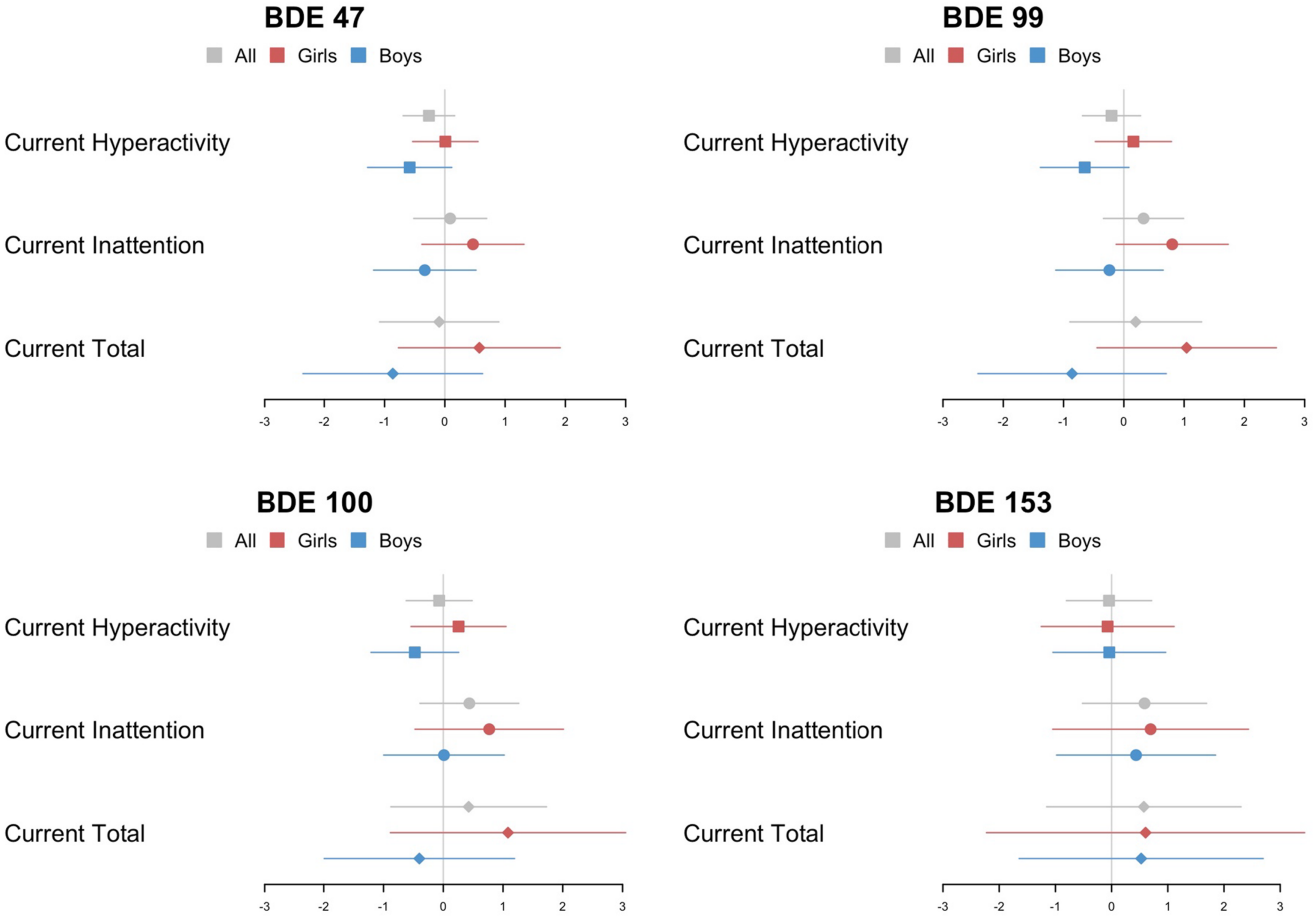
Point estimates (β) and 95% CIs from adjusted models examining continuous, natural-log-transformed plasma PBDE concentrations (ng/g lipid) in relation to the current ADHD rating scale hyperactivity, inattention, and total scores using GEE model (*n*_all _= 219, *n*_girls _= 121, *n*_boys _= 98). PBDE, polybrominated diphenyl ether; ADHD, attention deficit/hyperactivity disorder; GEE, generalized estimating equations.

As shown in Figure 3, the association between cord plasma PBDE concentration and ADHD-RS scores were close to null in GEE models using a combined sample of boys and girls, with sex included as a covariate. However, sex-stratified models demonstrated opposite trends; a positive association between exposure and outcome among girls, but among boys, the association is negative or largely null. Specifically, we found a trend of increasing current inattention score (0.819 increment out of 27) with a unit increase in natural-log-transformed cord plasma BDE-99 among girls (*p* = 0.09), but no association observed for boys (*p* = 0.67). Similar patterns are observed for the other three congeners (BDE-47, BDE-100, and BDE-153). Additional details describing the effect estimates of exposure-outcome from the GEE models are provided in Supplementary Table S1. In the sensitivity analyses, we did not observe that PBDE exposure trajectories, including postnatal exposure, were associated with symptoms of ADHD (results not shown). Compared to the regression results using lipid-standardized PBDE, no meaningful differences were observed from the results using wet-weight PBDEs with cord blood cholesterol and cord blood triglycerides as covariates.

### 3.4. Secondary and sensitivity analyses

**Figure 4:**
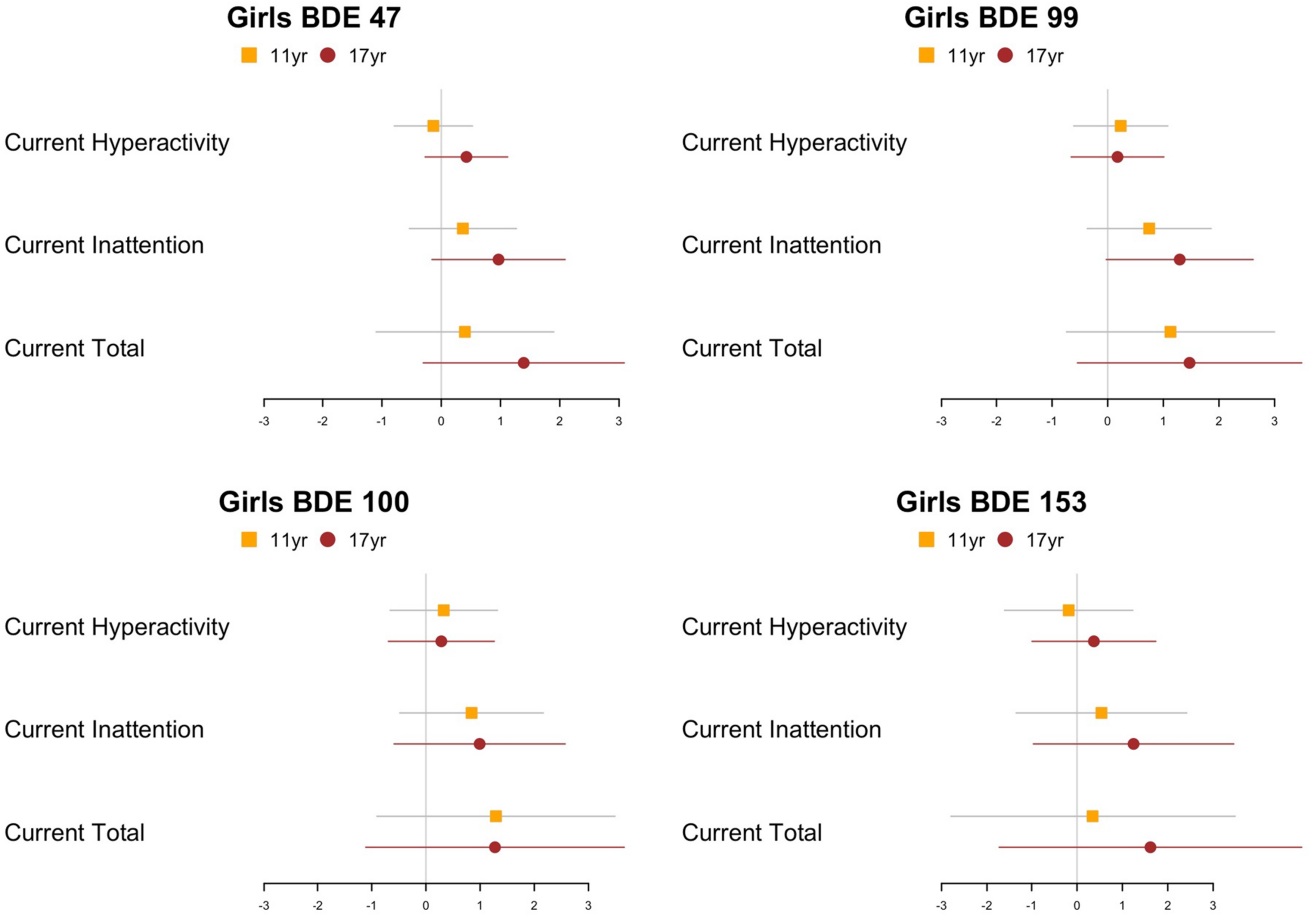
Point estimates (β) and 95% CIs from adjusted models examining continuous, natural-log- transformed plasma PBDE concentrations (ng/g lipid) in relation to the ADHD rating scale hyperactivity, inattention, and total scores of different samples (11 or 17 years) using linear model at each time point (*n*_11year _= 106, *n*_17year _= 69). PBDE, polybrominated diphenyl ether; ADHD, attention deficit/hyperactivity disorder.

Results from linear regression models examining each timepoint separately were similar to the GEE models. We found positive associations between PBDE exposure and ADHD inattention among girls but not boys. In general, among girls with ADHD measures at approximately 17 years of age, there is a stronger association between prenatal exposure to PBDE and parent-reported ADHD symptoms among girls with ADHD measures at approximately 17 years than 11 years (Figure 4), with a significant association observed for inattention symptoms (*p* = 0.05). Additional details describing the effect estimates are provided in Supplementary Table S2. In addition, the results of the GEE model examining the “worst ever” ADHD-RS score as the outcome are similar to the results of the “current” ADHD-RS score (Supplementary Figure S2).

## 4. Discussion

The objective of our study was to assess the association between prenatal exposure to PBDEs and attention problems assessed during adolescence, with the *a priori* hypothesis based on previous reports that child sex may modify this association (36). We observed a qualitative interaction between child sex and cord plasma penta-BDE concentrations (BDE-47, 99, and 100) on ADHD-RS scores, with divergent effects among boys and girls. Our results suggest that exposure to penta-BDE during pregnancy may have a long-lasting and sex-specific impact on adolescent neurodevelopment, specifically in the inattention subscale.

Previous epidemiologic studies have shown that prenatal exposure to PBDE is associated with attention problems at 3–7 years of age (24) and attention and executive function at 9–12 years of age (14), suggesting prenatal exposure to PBDE may affect children's neurodevelopment throughout early and mid-childhood, with effects observed into the adolescent years. Previous studies suggest that early-life exposure to endocrine-disrupting chemicals such as PBDEs may affect children's attention problems differently depending on the child's biological sex (37, 38). Given that here, PBDE exposure levels are similar by sex, it is possible that sex differences in biological activity, such as alterations in thyroid hormone function (39) and alterations in the levels of circulating sex hormones (40) in relation to PBDE exposure, may account for some of the observed variation. Indeed, some recent studies also found sex differences in the impact of prenatal exposure to PBDE on various measures of attention and executive function (14, 41) while others showed no detectable effect measure modification by sex (24, 26). It is possible that individual studies may have been under-powered to detect sex-specific associations, potentially due to relatively small sample sizes. In addition, the symptoms and prevalence of ADHD have been shown to vary by children’s sex, with boys typically endorsing more ADHD symptoms and some ADHD subtypes based on parents’ reports (42). There is also evidence that boys are more likely than girls to be diagnosed with ADHD and that differences in the expression of ADHD symptoms by child gender (not necessarily biological sex) may account for some of the differential prevalence (43). The sex-dependent biases among parental perceptions of ADHD symptoms in children may also be attributed to pro-social behaviors, in that girls’ symptoms of ADHD might be masked more efficiently by their positive social behaviors than that of boys’; thus, girls are less likely to be diagnosed with ADHD than boys (44).

While the mechanisms underlying prenatal PBDE neurotoxicity are not elucidated, expanding evidence supports disrupted maternal thyroid functioning during pregnancy and/or infancy as one potential mechanism. Due to their structural similarity, thyroid hormones are often regarded as targets of PBDEs (45). Previous papers showed that the thyroid regulatory system may be sensitive to disruption by PBDEs during both the pre- and postnatal periods. For example, in the study of 397 newborns at the University Hospital Centre of Sherbrooke, researchers found negative associations between cord plasma PBDEs and maternal total T4, free T3, and cord plasma free T4 (46), and the Healthy Pregnancy, Healthy Baby (HPHB) Study observed an association between PBDE measured in placenta tissue with altered thyroid hormones measured in infants (39). In addition, a recent paper in the same study population showed that children exposed to higher levels of PBDE are likely to have altered thyroid hormone levels in early childhood (47). The maternal and infant thyroid hormone is important for neurodevelopment because the thyroid hormone during gestation is involved in the neural migration, differentiation, myelination, and synaptogenesis of offspring (48). Studies have shown that maternal hypothyroxinemia, defined as low free T4 given normal thyroid-stimulating hormone (TSH), during gestation is positively associated with the incidence of inattention symptoms at various ages during childhood (49–51). Further, Simic et al. (52) showed that reduced levels of thyroid hormone in the neonatal period are associated with subsequent deficient attentional outcomes among preterm infants.

Interestingly, the HPHB Study (39) showed that that accumulation of PBDE in placental tissue is associated with altered thyroid hormone function in infants differently depending on the child’s sex, providing a possible mechanism to explain the inconsistent trend in the impact of prenatal exposure to PBDE on adolescents’ attention problem by sex. The associations of PBDE exposure, thyroid hormone function, sex-dependency, and fetal neurodevelopment during the critical period of fetal growth suggest that the neurobehavioral effects of prenatal exposure to PBDEs may persist through mid–late adolescence in a sex-specific manner. In addition to thyroid hormone disruption, there are other possible mechanisms through which prenatal PBDE exposure may disrupt child neurodevelopment, including direct action on brain development and oxidative stress (8). However, these competing hypotheses have been under-studied to date.

Our study has several strengths and weaknesses. First, we accounted for the repeated measure of ADHD-RS score that occurs at either approximately 11 years old or 17 years old using the GEE model, which allowed us to include all observations. While this is a strength, it also introduced some analytic challenges, as the age range is relatively broad. Then, a subsequent stratified analysis by age group allowed us to assess the persistence of ADHD symptoms over time and to determine if early vs. late adolescence is a more sensitive window for the detection of symptoms. However, since not all participants have repeated outcome measures from the two age points, the discrepancy by age group we observed might be related to unmeasured sample differences. Moving forward, a longitudinal study with a single analysis group to assess the impact of gestational exposure to PBDE on neurodevelopmental trajectory will help understand this result. In addition, we relied on parents’ reports of inattention/hyperactivity symptoms; thus, future studies could also include teacher or self-report to capture adolescents’ ADHD symptoms more fully. Moreover, we used an error rate of *p*-value <0.20 to assess the significance of the interaction term given our limited statistical power; however, this threshold is liberal enough to generate the cost of more type I errors. Furthermore, as high proportions of PBDE values were below the detection limits and imputed in our analyses, this may have biased the results (53). Future studies with higher detection rates and utilizing advanced imputation methods will strengthen our results. In addition, we did not examine the mixture effects of PBDE since the goal of this study was to extend previous analyses (24) that have examined the effects of prenatal PBDE exposure congener-by-congener on attention and ADHD indicators in children at younger ages. However, as the exposure occurs in a mixture form, future studies utilizing mixture analysis examining the neurological impact of PBDE mixture will be necessary. Moreover, the study population was composed solely of either Black or Dominican women and their children, thus limiting the generalizability of the results. Finally, due to the small sample size, particularly in stratified models, many of our effect estimates have wide confidence intervals, often including the null. However, the direction of effect was relatively consistent, providing more confidence in our results. Future studies with a larger sample size are necessary to verify the effect estimate of prenatal exposure to PBDE on adolescents’ inattention symptoms and sex-specificity of the association.

## 5. Conclusion

In this study, we demonstrated preliminary evidence that sex may act as a potential effect modifier of the association between prenatal exposure to PBDE and symptoms of adolescent ADHD. Cord plasma PBDE concentrations were positively associated with inattention among girls, but not boys during mid or late adolescence, but notably the interaction effect was not significant. Thus, further study is required to replicate our findings in a larger and more diverse sample and to specifically test sex-specific effects of PBDE exposure on attentional processes. Our findings align with those of previous studies reporting the association between prenatal PBDE exposure and children's ADHD symptoms. Collectively, our study, in combination with prior research, suggests the effects of prenatal PBDE exposure may be sex-specific and persist through late adolescence, and emphasizes the need to follow children longitudinally to fully capture the impact of early-life exposure on life-long health and its dimorphism by sex.

## Data Availability

To protect research participants' privacy and confidentiality, data will be released only after approval of the request by study's steering committee as well as the requesting institution's IRB or equivalent body.
