# Supplementary material for: Prenatal exposure to polybrominated diphenyl ethers and inattention/hyperactivity symptoms in mid to late adolescents
Source: Front Epidemiol. 2023 Jun 21;3:1061234. doi: 10.3389/fepid.2023.1061234 (PMC10910905; doi:10.3389/fepid.2023.1061234)
Supplement: Supplementary file 1 [file Datasheet1.docx]

**Supplementary Materials**


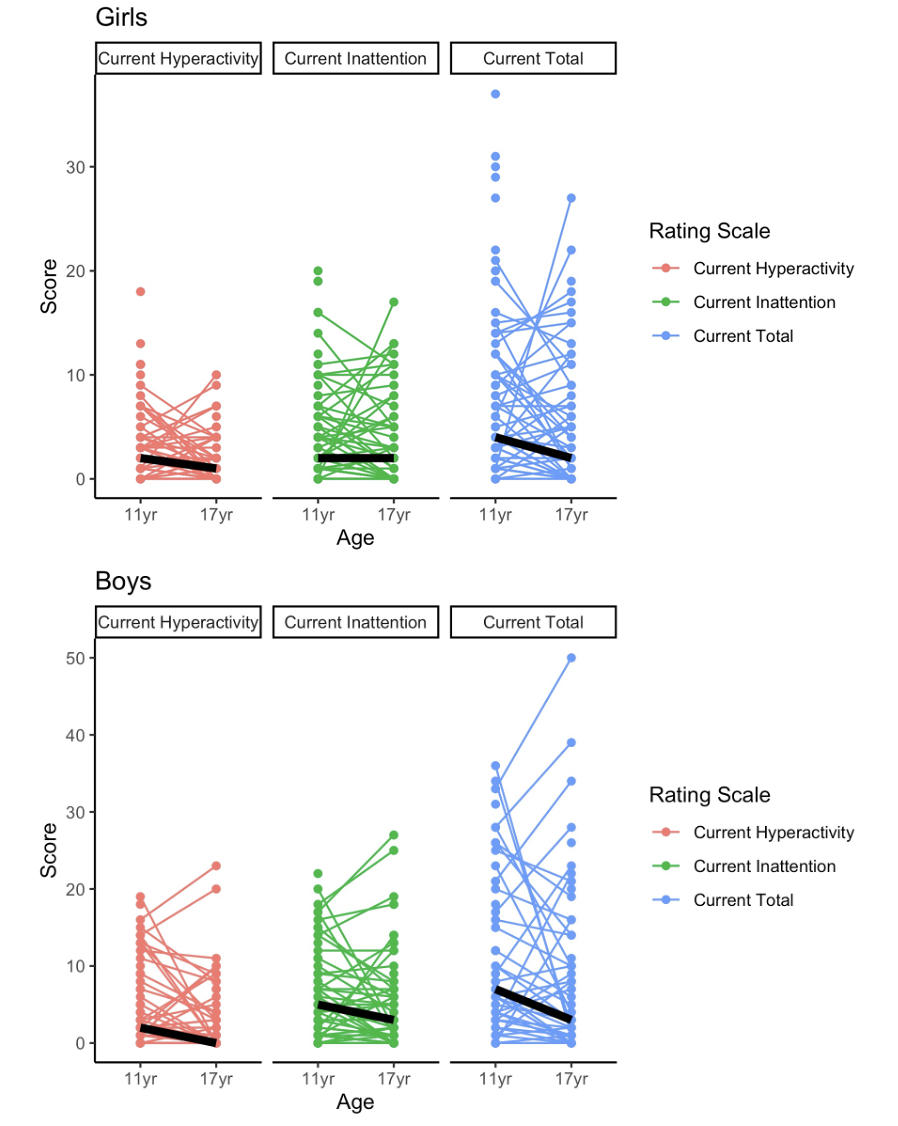


**Supplementary Figure S1**. Spaghetti plots of the change of ADHD rating scale score of children whose score were reported twice during the study period. Black solid lines indicate the change of median score from approximately 11 years (9-14) to 17 years (14-21) of age.


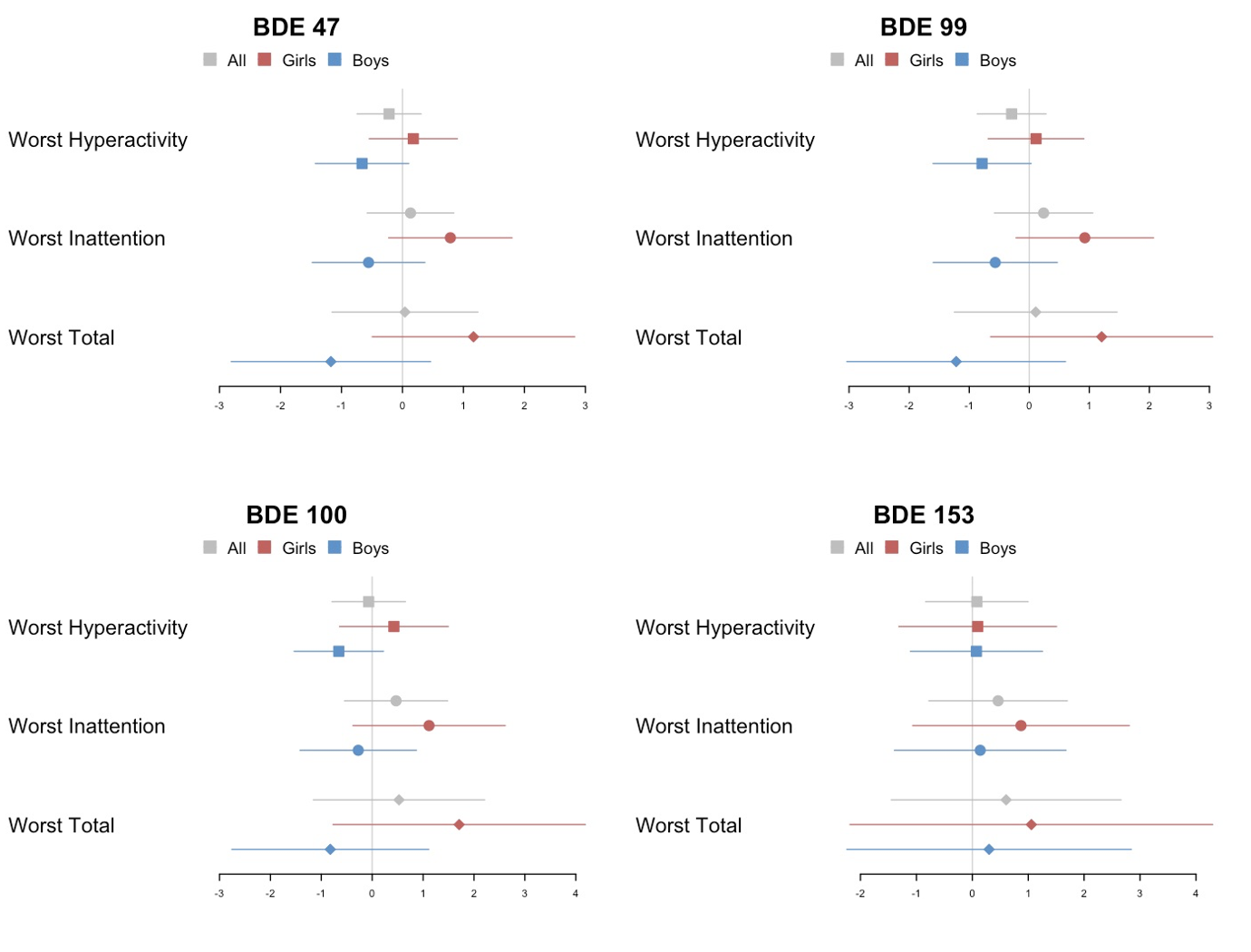


**Supplementary Figure S2**. Point estimates (ß) and 95% CIs from adjusted models examining continuous, log10- transformed plasma PBDE concentrations (ng/g lipid) in relation to the **worst** ADHD Rating Scale Hyperactivity, Inattention, and Total scores using GEE model.

|  |  | | |  | | |  | | |
| --- | --- | --- | --- | --- | --- | --- | --- | --- | --- |
|  | | | 11 yr  (n=187) | | | 17 yr  (n=134) | p-value | | |
| **Maternal characteristics** | |  | | | |  |  | | |
| Age (years) | | 24.94 ± 5.13 | | | | 24.84 ± 4.83 | 0.86 | | |
| Education | |  | | | |  |  | | |
| <High school | | 10 (5.35) | | | | 6 (4.48) | 0.94 | | |
| High school or GED | | 129 (68.98) | | | | 93 (69.4) |  |  |  |
| ≥ College degree | | 48 (25.67) | | | | 35 (26.12) |  |  |  |
| Married/living with partner | | 57 (30.48) | | | | 37 (27.61) | 0.66 | | |
| Race/ethnicity | |  | | | |  |  | | |
| African American | | 81 (43.32) | | | | 48 (35.82) | 0.22 | | |
| Dominican | | 106 (56.68) | | | | 86 (64.18) |  |  |  |
| Nonverbal intelligence | | 84.60 ± 13.26 | | | | 84.34 ± 12.94 |  | | |
| **Household characteristics** | |  | | | |  |  | | |
| Material hardship^a^ | | 76 (40.64) | | | | 49 (36.57) | 0.46 | | |
| Smoker in home | | 64 (34.22) | | | 41 (30.6) | | 0.54 | | |
|  | |  | | |  | |  | | |
| **Child Characteristics** | |  | | |  | |  | | |
| Birth weight (kg) | | 3.44 ± 0.54 | | | 3.43 ± 0.57 | | 0.94 | | |
| Gestational age (weeks) | | 39.37 ± 1.31 | | | 39.02 ± 4.37 | | 0.31 | | |
| Breastfed $\geq$ 12 weeks^b^ | | 60 (32.09) | | | 49 (36.57) | | 0.47 | | |
| Cord PBDE (ng/g lipid)^c^ | |  | | |  | |  | | |
| BDE 47 | | 13.73 ± 3.06 | | | 14.03 ± 3.10 | | 0.68 | | |
| BDE 99 | | 3.72 ± 2.46 | | | 3.71 ± 2.48 | | 0.89 | | |
| BDE 100 | | 2.96 ± 2.08 | | | 2.96 ± 2.14 | | 0.76 | | |
| BDE 153 | | 2.59 ± 1.72 | | | 2.58± 1.76 | | 0.83 | | |
| a: Self-reported inability to afford adequate access to food, clothing, or housing | | | | | | | | |  |
| b: Percent of ‘Yes’ for breastfeed $\geq$ 12 weeks | | | | | | | | |  |
| c: geometric mean |  | | |  | | |  |  |  |
|  |  | | |  | | |  |  |  |

**Supplementary Table S1.** Characteristics of participants by age group (n_11yr_ = 187, n_17yr_ = 134) Values are Mean ± SD or n (%). One-way ANOVA tests or Chi-squared tests were used to test for differences in means between age groups. All characteristics are measured at baseline (pregnancy/delivery) unless otherwise noted

|  |  |  | |  |  |
| --- | --- | --- | --- | --- | --- |
| **Group** | **RS** | **BDE 47** | **BDE 99** | **BDE 100** | **BDE 153** |
| All | Current Hyperactivity | -0.253 (-0.677 , 0.171) | -0.189 (-0.669 , 0.291) | -0.061 (-0.611 , 0.49) | -0.034 (-0.792 , 0.724) |
|  | Current Inattention | 0.085 (-0.516 , 0.685) | 0.33 (-0.333 , 0.992) | 0.433 (-0.393 , 1.258) | 0.589 (-0.516 , 1.694) |
|  | Current Total | -0.087 (-1.068 , 0.893) | 0.215 (-0.873 , 1.303) | 0.429 (-0.871 , 1.729) | 0.589 (-1.141 , 2.318) |
| Girls | Current Hyperactivity | 0.024 (-0.51 , 0.557) | 0.182 (-0.44 , 0.804) | 0.267 (-0.517 , 1.052) | -0.049 (-1.218 , 1.121) |
|  | Current Inattention | 0.472 (-0.363 , 1.307) | 0.819 (-0.101 , 1.738) | 0.779 (-0.455 , 2.014) | 0.711 (-1.025 , 2.447) |
|  | Current Total | 0.593 (-0.725 , 1.912) | 1.082 (-0.385 , 2.549) | 1.105 (-0.842 , 3.052) | 0.645 (-2.162 , 3.451) |
| Boys | Current Hyperactivity | -0.586 (-1.28 , 0.107) | -0.653 (-1.383 , 0.077) | -0.492 (-1.219 , 0.234) | -0.048 (-1.051 , 0.955) |
|  | Current Inattention | -0.353 (-1.195 , 0.489) | -0.258 (-1.144 , 0.628) | -0.022 (-1.027 , 0.983) | 0.425 (-0.99 , 1.839) |
|  | Current Total | -0.889 (-2.364 , 0.587) | -0.881 (-2.434 , 0.672) | -0.45 (-2.031 , 1.131) | 0.512 (-1.663 , 2.686) |

**Supplementary Table S2.** Point estimates (ß) and 95% CIs from Figure 3 (n_all_ = 219, n_girls_=121, n_boys_=98).

| **Group** | **RS** | **BDE 47** | **BDE 99** | **BDE 100** | **BDE 153** |
| --- | --- | --- | --- | --- | --- |
| Girls at 11yr | Current Hyperactivity | -0.132 (-0.791 , 0.527) | 0.234 (-0.611 , 1.08) | 0.329 (-0.661 , 1.319) | -0.183 (-1.596 , 1.23) |
|  | Current Inattention | 0.365 (-0.538 , 1.269) | 0.746 (-0.368 , 1.859) | 0.842 (-0.483 , 2.166) | 0.539 (-1.341 , 2.418) |
|  | Current Total | 0.4 (-1.098 , 1.899) | 1.129 (-0.741 , 2.999) | 1.292 (-0.904 , 3.488) | 0.344 (-2.794 , 3.483) |
| Girls at 17yr | Current Hyperactivity | 0.425 (-0.271 , 1.121) | 0.176 (-0.657 , 1.009) | 0.284 (-0.693 , 1.26) | 0.372 (-0.991 , 1.735) |
|  | Current Inattention | 0.967 (-0.156 , 2.09) | 1.295 (-0.025 , 2.615) | 0.991 (-0.588 , 2.569) | 1.246 (-0.961 , 3.453) |
|  | Current Total | 1.392 (-0.303 , 3.087) | 1.471 (-0.544 , 3.487) | 1.274 (-1.111 , 3.659) | 1.618 (-1.714 , 4.95) |

**Supplementary Table S3.** Point estimates (ß) and 95% CIs from Figure 4 (n_11yr_ = 106, n_17yr_ = 69).
